# Effect of the CO_2_ laser on enamel surface roughness after debonding ceramic brackets and the application of nanohydroxyapatite: *in-vitro* study

**DOI:** 10.3389/fdmed.2026.1912248

**Published:** 2026-09-11

**Authors:** A. Mar-Ramírez, L. E. Rodríguez Vilchis, R. J. Scougall-Vilchis, M. Nagayama, R. Contreras Bulnes

**Affiliations:** 1 Department of Orthodontics and Preventive Dentistry, Center of Research and Advanced Studies in Dentistry, School of Dentistry, Autonomous University of México State, Toluca, México State, México; 2 Department of Oral Pathology, School of Dentistry, Asahi University, Gifu, Japan

**Keywords:** ceramic brackets, CO_2_ laser, nanohydroxyapatite paste, shear bond strength, surface roughness

## Abstract

**Objective:**

Evaluate the effect of the CO_2_ laser on enamel surface roughness after debonding ceramic brackets and applying nanohydroxyapatite.

**Methodology:**

Sixty extracted premolars were randomly assigned to four groups (*n* = 15): G1, G2, G3, G4. Ceramic brackets were bonded to the buccal surface for G1 and G2 groups. Before debonding, the CO_2_ laser group (G2) was irradiated with a power of 6 W for 5 s; shear bond strength was calculated using a universal testing machine and the adhesive remnant index with a stereomicroscope. Enamel roughness for the four groups was assessed using a profilometer; then, each group was subdivided for the application of nanohydroxyapatite paste and stored in saliva or deionized water. The surfaces were re-evaluated after 15, 30, and 60 days; the Friedman and Nemenyi *post-hoc* tests were used for statistical analysis, while Kruskal–Wallis and Dunn's multiple comparison *post-hoc* tests with Holm's correction adjustment to compare between groups.

**Results:**

The group irradiated with CO_2_ laser significantly reduced bond strength and roughness parameters upon debonding compared to the bracket group (*p* < 0.001). No significant differences were observed in roughness reduction between the group treated with the CO_2_ laser and the application of nanohydroxyapatite paste; however, the surface roughness decreased in the bracket group G1b.

**Conclusions:**

CO_2_ laser irradiation reduced the force required for debonding ceramic brackets and minimized enamel surface roughness compared with conventional debonding. This contributes to the development of enamel-preserving orthodontic treatments; however, this property does not improve when applying nanohydroxyapatite paste.

## 1 Introduction

Fixed orthodontic appliances aim to achieve an optimal occlusion that allows a balanced structural and functional adjustment, periodontal health, and aesthetic harmony of the orofacial region (1, 2). Notably, aesthetic aspects play a very important role in orthodontic treatment. Although ceramic brackets were introduced in 1980 (3), they continue to play a key role today, especially in the anterior region, where less visibility is desired (4). In addition, these brackets offer advantages such as biocompatibility, optimal esthetics, resistance to chemical and thermal changes, and adequate bond strength, while maintaining the biomechanical performance required by the clinician (4, 5). Brackets are composed of aluminum oxide ([Al_2_] [O_3_]) and were classified by Michel L. Swartz according to their structure: polycrystalline and monocrystalline ceramic brackets, with mechanical or chemical retention (6). The difference between both types lies in the optical clarity since polycrystalline ceramic brackets reflect light, giving rise to a certain degree of opacity (1, 7, 8).

Once orthodontic treatment has been completed, the appliance must be removed without damaging the tooth structure; however, enamel and bracket fractures are common during conventional debonding because the shear bond strength (SBS) exceeds 10 MPa (9). It is estimated that between 5 and 20 µm of enamel is lost, leaving rough surfaces that make proper cleaning difficult, promoting the accumulation of plaque and bacteria that will favor demineralization (10, 11). Recent methods, such as nanohydroxyapatite (nHAp) application, have been studied to achieve remineralization of the enamel surface (12). The nHAp is a form of synthetic apatite with chemical and structural characteristics similar to dental hard tissue, its 20 nm particles promote the rearrangement of calcium phosphate ions in the form of hydroxyapatite, leading to the formation of a thin but firmly adherent layer on the tooth surface, significantly increasing surface hardness and remineralization (12–14).

Several methods have been suggested to facilitate the debonding of ceramic brackets by promoting a reduction in shear bond strength (15). Four approaches stand out: mechanical debonding, ultrasonic debonding, electrothermal debonding, and laser debonding (16, 17). Laser-assisted bracket removal has been reported to achieve painless debonding without damaging pulp and with a reduction in removal time between 1 and 5 s. Various laser devices have been studied for debonding ceramic brackets including carbon dioxide (CO_2_) diode, erbium-doped yttrium aluminum garnet (Er:YAG), and neodymium-doped yttrium aluminum garnet (Nd:YAG) (15, 18).

Studies have concluded that the mode of action for debonding is based on the thermal softening of the resin adhesive caused by laser-induced heat, which is transmitted from the bracket to the resin. The latter can occur by thermal ablation, and photoablation (18–21). The CO_2_ laser is available primarily at a wavelength of 10.6 μm, which is highly absorbed by water, minerals such as hydroxyapatite, and ceramic materials, making it a suitable instrument for the treatment of ceramic surfaces and for the debonding of ceramic brackets (18, 22, 23).

An effective way to assess the different degrees of enamel damage after debonding ceramic brackets and to analyze surface morphology is the profilometer, which analyzes characteristics such as profile height and roughness at the nanometer level (21, 24).

Various debonding methods, enamel cleaning and polishing techniques, as well as remineralizing agents, have been studied. However, to date, no method has demonstrated the ability to preserve the integrity of enamel without causing any degree of damage, nor to completely restore its physicochemical properties to its original pre-treatment condition. Therefore, we seek to put alternative methods forward that enable the enamel surface repair after debonding, by focusing on less invasive and more comfortable techniques for the patient.

Therefore, the aim of this study is to evaluate the use of a CO_2_ laser for debonding ceramic brackets together with the effect of the subsequent application of nanohydroxyapatite on the roughness of the enamel surface. The analysis focuses on SBS and enamel surface characterization, followed by the evaluation of nHAp effects on enamel roughness under different conditions and time points.

## 2 Materials and methods

This study was carried out under the Declaration of Helsinki and approved by the Research Ethics Committee of the Center for Research and Advanced Studies in Dentistry of the School of Dentistry at the Autonomous University of the State of Mexico with registration number: (CEICIEAO-2023-009).

### 2.1 Sample preparation

Sound human premolars extracted for orthodontic reasons were included, free of caries, cracks, restorations, or developmental defects. The teeth were cleaned under running water to remove blood and tissue debris, polished with fluoride-free prophylactic paste using a rubber cup at low speed, and stored in distilled water in a 37 °C incubator for a maximum of three months until testing. Specimens were then distributed randomly allocated into four groups (*n* = 15) using a computer-generated sequence with the RAND() function in Microsoft Excel to minimize selection bias (25): G1 (bracket), G2 (bracket laser), G3 (polished enamel), and G4 (healthy enamel).

The buccal surface was conditioned following the same protocol as employed in the G1 and G2 groups. Initially, 35% phosphoric acid (Ultra-etch, Ultradent Products Inc., South Jordan, UT, USA) was applied for 15 s. Subsequently, the surface was rinsed with water for 20 s to ensure thorough removal of the etchant, followed by air-drying for 5 s.

A thin layer of adhesive (3M Unitek, Monrovia, CA, USA) was applied to the enamel surface, dispersed using oil-free air, and subsequently light-cured for 10 s.

In group G3, the buccal surface was polished with aluminum oxide tips (Onegloss, Shofu Inc., Kyoto, Japan) by applying gentle pressure for 10 s in conjunction with the use of diamond polish (Diamond polish, Ultradent Products Inc., South Jordan, UT, USA).

### 2.2 Bracket bonding

Thirty polycrystalline ceramic brackets (Clarity Advanced, 3M Unitek) were bonded to the center of the buccal surface of the teeth in groups G1 and G2 using Transbond Plus Color Change Adhesive (3M Unitek). Excess resin was removed with the tip of a dental explorer. The adhesive was light-cured for 6 s on the mesial and distal sides of the bracket using a light-emitting diode (LED) curing unit with an output intensity of 1600 mW/cm^2^ (Ortholux, 3M Unitek).

### 2.3 CO_2_ laser irradiation and shear bond strength test

Prior to the debonding procedure, samples from the G2 group were irradiated using a CO_2_ laser (The Yoshida Dental MFG, CO., LTD, Tokyo, Japan) operating in continuous mode at a wavelength of 10.6 μm and a power of 6 W.

The light was applied perpendicularly at 5 mm from the bracket surface, performing horizontal sweeping movements for 5 s. Immediately following irradiation, a chisel-shaped blade was positioned at the enamel/bracket base interface to perform the debonding procedure.

SBS testing was performed using a universal testing machine (EZ Graph, Shimadzu Corporation, Kyoto, Japan), applying a force in the occluso-gingival direction to produce detachment at a crosshead speed of 1 mm/min, the resulting values were recorded in megapascals (MPa).

### 2.4 Evaluation of the adhesive remnant index (ARI)

A stereomicroscope at 10× magnification (VE-S5C, Velab, Guadalajara, Jalisco, México) was used to quantify the amount of residual resin adhered to the enamel surface.

Residual resin was removed using a 12-blade tungsten carbide bur (C375R, Jota AG, Rüthi, Switzerland) and polished with a OneGloss tip (Shofu Inc., Kyoto, Japan) and diamond paste for 10 s. ARI scoring was performed by a single experienced observer, without knowing the teeth group allocation, using standardized criteria. Consistency was ensured through repeated evaluation of the samples.

### 2.5 Enamel surface assessment

The buccal surface of each tooth was sectioned at a thickness of 4 mm using a micromotor with 0.20 mm diamond cutting discs.

Samples from the four groups were evaluated with a profilometer (Mitutoyo Surftest SJ-301, Tokyo, Japan), using surface roughness parameters Ra, Rq and Rz recorded in micrometers. Three distinct locations at the center of each specimen were measured, and the values were averaged to produce a single representative value per tooth. In total, 15 profilometric measurements were acquired per experimental condition. A diamond tip of 5 μm radius was employed, operating with a cutoff value (*λ*c) of 0.08 mm, a cross-sectional length of 0.5 mm, a scanning speed of 0.25 mm/sec, and a Gaussian filter algorithm. These parameters were selected according to internationally accepted standards and recommendations for contact surface profilometry (ISO 21920-2:2021; ISO 21920-3:2021), which replaced the earlier ISO 4287/4288 widely cited in biomaterials research (26, 27).

### 2.6 Application of the nanohydroxyapatite paste

**Figure 1:**
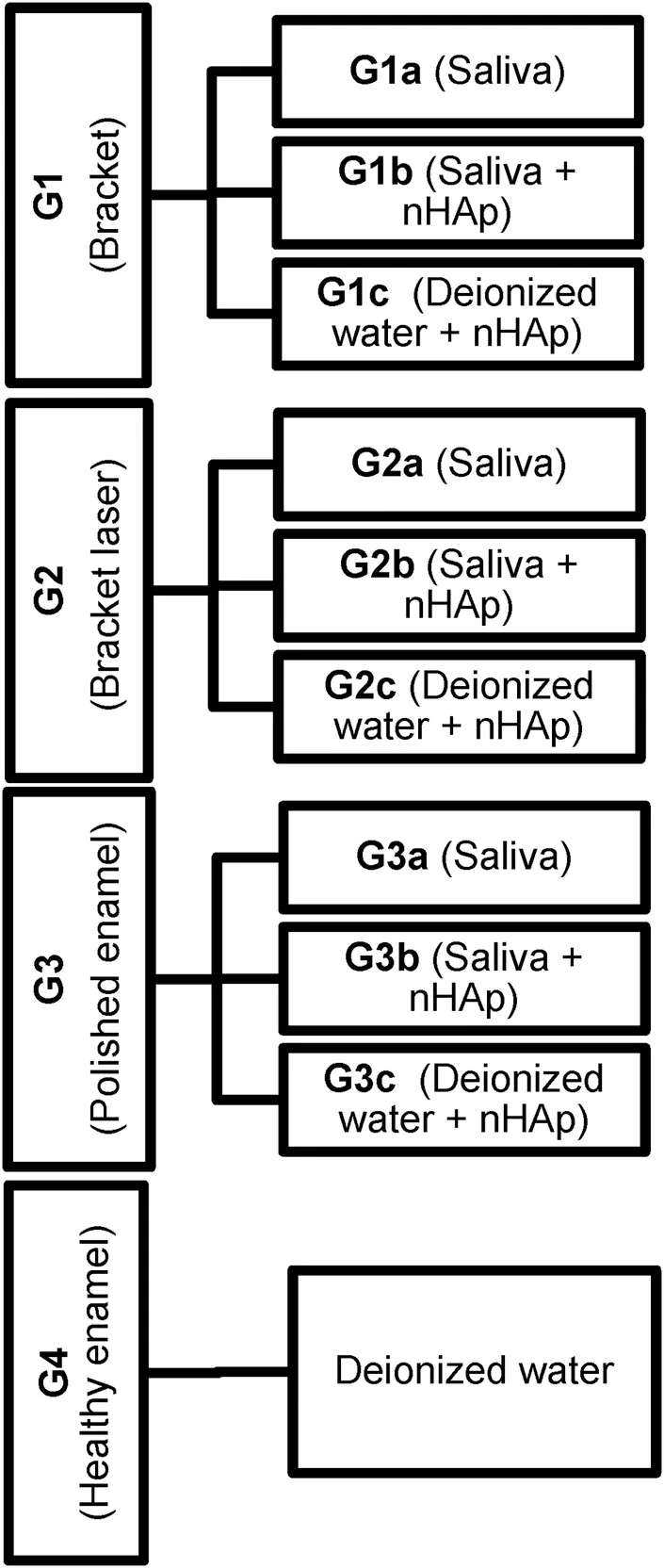
Sample distribution into groups according to the treatment performed.

Groups G1, G2, and G3 were further subdivided into three experimental subgroups *n* = 5, (Figure 1). One group was stored untreated in artificial saliva (a), while the remaining groups received nanohydroxyapatite paste (Apagard, Sangi Co., Ltd., Japan) for a period of two months and were stored in artificial saliva (b) and deionized water (c), respectively. The artificial saliva (Viarden S.A. de C.V., México) was composed of mineral salts (Na⁺, K⁺, F⁻, Cl^2^⁻, Mg^2^⁺, Ca^2^⁺, PO₄^2^⁻), xylitol, enzymes, and flavoring agents, according to the manufacturer's formulation. The enamel surface was brushed twice daily for 3 min using an electric toothbrush, with a bean-sized amount of nanohydroxyapatite paste applied during each session and rinsed with distilled water to ensure complete removal of the paste. The use of an electric toothbrush ensured a standardized brushing motion and minimized variability in force application, and the operator was previously trained to keep handling consistently across all specimens. All specimens were stored in an incubator at 37 °C, with artificial saliva and deionized water refreshed every 48 h. Surface evaluations were conducted at 15, 30, and 60 days using a profilometer.

### 2.7 Scanning electron microscopy (SEM) evaluation

The enamel surfaces were examined with a scanning electron microscope (JEOL JSM-6510LV, JEOL Ltd., Akishima, Tokyo, Japan) in three representative sections located in the center of the tooth crown. Fields were selected within the area previously covered by the bonded bracket, ensuring that the evaluation focused specifically on the region subjected to bonding and debonding procedures. SEM observations were performed to qualitatively describe representative morphological features of the enamel surface. Micrographs were obtained at magnifications of ×200, ×1000, and ×1500, with a field of view of approximately 10 µm × 10 µm. Images were acquired under low-vacuum conditions and a working distance of approximately 10 mm.

### 2.8 Statistical analysis

The collected numerical data were analyzed by the Statistical Software SPSS version 25.0 (IBM, Armonk, New York, USA) and Python 3.13 (Python Software Foundation PSF).

Data normality was assessed using the Shapiro–Wilk test, while the comparison of SBS values between the G1 and G2 groups was conducted using Student's t-test. The Mann–Whitney U test was employed to analyze ARI scores. Surface roughness parameters across 0, 15, 30, and 60 days were evaluated using the Friedman tests and Nemenyi *post-hoc* tests (Nemenyi-Damico-Wolfe); for changes between groups, Kruskal–Wallis and Dunn's multiple comparison *post-hoc* tests with Holm's correction adjustment method were used. To further confirm the adequacy of the sample size, a *post hoc* power analysis was conducted using G*Power software (version 3.1.9.7, Heinrich-Heine University, Düsseldorf, Germany).

## 3 Results

### 3.1 SBS

The group irradiated with the CO_2_ laser (G2) exhibited a statistically significant reduction in bond strength during debonding when compared to the bracket group (G1) (*p* < 0.001), as shown in Table 1.

**Table 1:** Shear bond strength (MPa).

Group	Mean (SD) MPa
G1	22.36 (6.11)a
G2	10.96 (5.51)b

Different letters: statistically significant difference (*p* < 0.05).

### 3.2 ARI

The Mann–Whitney U test showed a statistically significant difference in the location of bond failure between the two groups (*p* < 0.044), as presented in Table 2.

**Table 2:** Frequency distribution of the ARI Index.

Group	% ARI Index				
	0	1	2	3	Total
G1	13	27	20	40	100a
G2	40	40	0	20	100b

Different letters: statistically significant difference (*p* < 0.05).

### 3.3 Roughness

Statistically significant differences in roughness values were observed prior to paste application of groups G2, G3 and G4 compared to group G1 (*p* < 0.001). In contrast, no significant differences were observed among Groups G2, G3, and G4 (*p* ≥ 0.605) (Table 3).

**Table 3:** Results of enamel roughness prior to nHAp paste treatment.

Group	Ra	Rq	Rz
G1	0.210 ± 0.022a	0.278 ± 0.038a	1.210 ± 0.213a
G2	0.130 ± 0.011b	0.165 ± 0.017b	0.732 ± 0.110b
G3	0.131 ± 0.015b	0.166 ± 0.017b	0.727 ± 0.112b
G4	0.138 ± 0.026b	0.178 ± 0.032b	0.728 ± 0.124b

Significance level ≤0.05, Different letters: statistically significant difference.

Group G2b showed a significant within-group reduction in Ra and Rq after 30 days of toothbrushing with nHAp paste, in contrast, a decrease in Rz was observed only when compared with the group G2a. Group G1b showed a significant between-group reduction in surface roughness with Ra decreasing after 15 days, and Rq, Rz showing further reduction after 60 days of toothbrushing (Table 4).

**Table 4:** Descriptive statistics and statistical analysis results of enamel roughness at multiple time points among the groups.

Roughness parameter	Group	T0	T15	T30	T60
Ra	G1a	0.212 ± 0.010 A,a	0.185 ± 0.008 A,B,a	0.171 ± 0.016 B,a	0.172 ± 0.012 B,a,b
	G1b	0.206 ± 0.034 A,a	0.175 ± 0.024 B,C,a	0.175 ± 0.007 B,a,b	0.158 ± 0.009 C,a
	G1c	0.213 ± 0.014 A,a	0.192 ± 0.007 B,a	0.246 ± 0.028 A,b	0.224 ± 0.013 A,b
	G2a	0.129 ± 0.004 A,a	0.132 ± 0.006 A,a	0.177 ± 0.017 B,a	0.143 ± 0.011 A,a
	G2b	0.126 ± 0.009 A,a	0.129 ± 0.014 A,B,a	0.139 ± 0.005 B,C,b	0.148 ± 0.009 C,a
	G2c	0.136 ± 0.015 A,a	0.141 ± 0.015 A,B,a	0.169 ± 0.011 B,C,a	0.221 ± 0.007 C,b
	G3a	0.124 ± 0.004 A,a	0.116 ± 0.008 A,a	0.138 ± 0.006 B,a	0.128 ± 0.001 A,B,a
	G3b	0.133 ± 0.008 A,B,a	0.127 ± 0.002 A,a	0.150 ± 0.009 C,a	0.140 ± 0.004 B,C,a,b
	G3c	0.136 ± 0.014 A,a	0.159 ± 0.007 B,b	0.155 ± 0.009 A,B,a	0.158 ± 0.005 A,B,b
Rq	G1a	0.280 ± 0.018 A,a	0.240 ± 0.019 A,B,a	0.217 ± 0.023 B,a	0.212 ± 0.022 B,a
	G1b	0.277 ± 0.058 A,a	0.220 ± 0.030 B,C,a	0.219 ± 0.019 A,B,a	0.199 ± 0.011 C,a
	G1c	0.277 ± 0.025 A,a	0.234 ± 0.009 B,a	0.308 ± 0.033 A,a	0.273 ± 0.014 A,b
	G2a	0.163 ± 0.009 A,a	0.171 ± 0.009 A,a	0.231 ± 0.025 B,a,	0.181 ± 0.012 A,a
	G2b	0.158 ± 0.014 A,a	0.160 ± 0.016 A,a	0.170 ± 0.007 A,b	0.191 ± 0.012 B,a
	G2c	0.175 ± 0.019 A,B,a	0.174 ± 0.019 A, a	0.206 ± 0.014 B,C,a	0.271 ± 0.007 C,b
	G3a	0.158 ± 0.006 A,a	0.147 ± 0.010 A,a	0.176 ± 0.008 B,a	0.160 ± 0.002 A,B,a
	G3b	0.167 ± 0.009 A,B,a	0.163 ± 0.005 A,a	0.192 ± 0.02° C,a	0.183 ± 0.008 B,C,a
	G3c	0.171 ± 0.016 A,a	0.205 ± 0.013 B,C,b	0.192 ± 0.010 A,B,a	0.211 ± 0.006 C,b
Rz	G1a	1.206 ± 0.077 A,a	1.117 ± 0.151 A,B,a	1.020 ± 0.129 A,B,a,b	0.927 ± 0.114 B,a
	G1b	1.226 ± 0.246 A,a	0.901 ± 0.162 B,a	0.889 ± 0.171 B,a	0.816 ± 0.072 B,a
	G1c	1.199 ± 0.152 A,a	0.962 ± 0.130 B,a	1.269 ± 0.100 A,b	1.132 ± 0.054 A,b
	G2a	0.713 ± 0.052 A,a	0.836 ± 0.075 A,B,a	1.029 ± 0.113 C,a	0.861 ± 0.058 B,C,a
	G2b	0.698 ± 0.098 A,a	0.690 ± 0.098 A,a	0.718 ± 0.022 A,B,b	0.829 ± 0.053 B,a
	G2c	0.784 ± 0.088 A,a	0.723 ± 0.087 A,a	0.810 ± 0.108 A,a,b	1.109 ± 0.048 B,b
	G3a	0.699 ± 0.026 A,B,a	0.676 ± 0.051 A a	0.782 ± 0.027 B,a	0.699 ± 0.033 A,B,a
	G3b	0.758 ± 0.050 A,a	0.735 ± 0.061 B,a,b	0.761 ± 0.063 C,a	0.795 ± 0.061 D,a,b
	G3c	0.723 ± 0.031 A,a	0.864 ± 0.073 B,b,	0.824 ± 0.104 A,B,a	0.890 ± 0.091 B,b

T0 no treatment, T15 after 15 days of nHAp application, T30 after 30 days of nHAp application, T60 after 60 days of nHAp application.

Different uppercase letters within the same row indicate statistically significant differences between groups at different time points (*p* < 0.05).

Different lowercase letters within the same column indicate significant statistically differences within groups at the same time point (*p* < 0.05).

Friedman tests revealed significant differences in surface roughness parameters within the experimental groups. Most analyses demonstrated moderate to large effect sizes (Kendall's W), with *post hoc* statistical power exceeding 98%. For intergroup comparisons, Kruskal–Wallis analyses consistently yielded large effect sizes (*ε*^2^ = 0.64–0.85), while pairwise comparisons conducted using Dunn's test with Holm-adjusted *p*-values confirmed robust differences among groups, further supporting the adequacy of the selected sample size.

### 3.4 SEM

**Figure 2:**
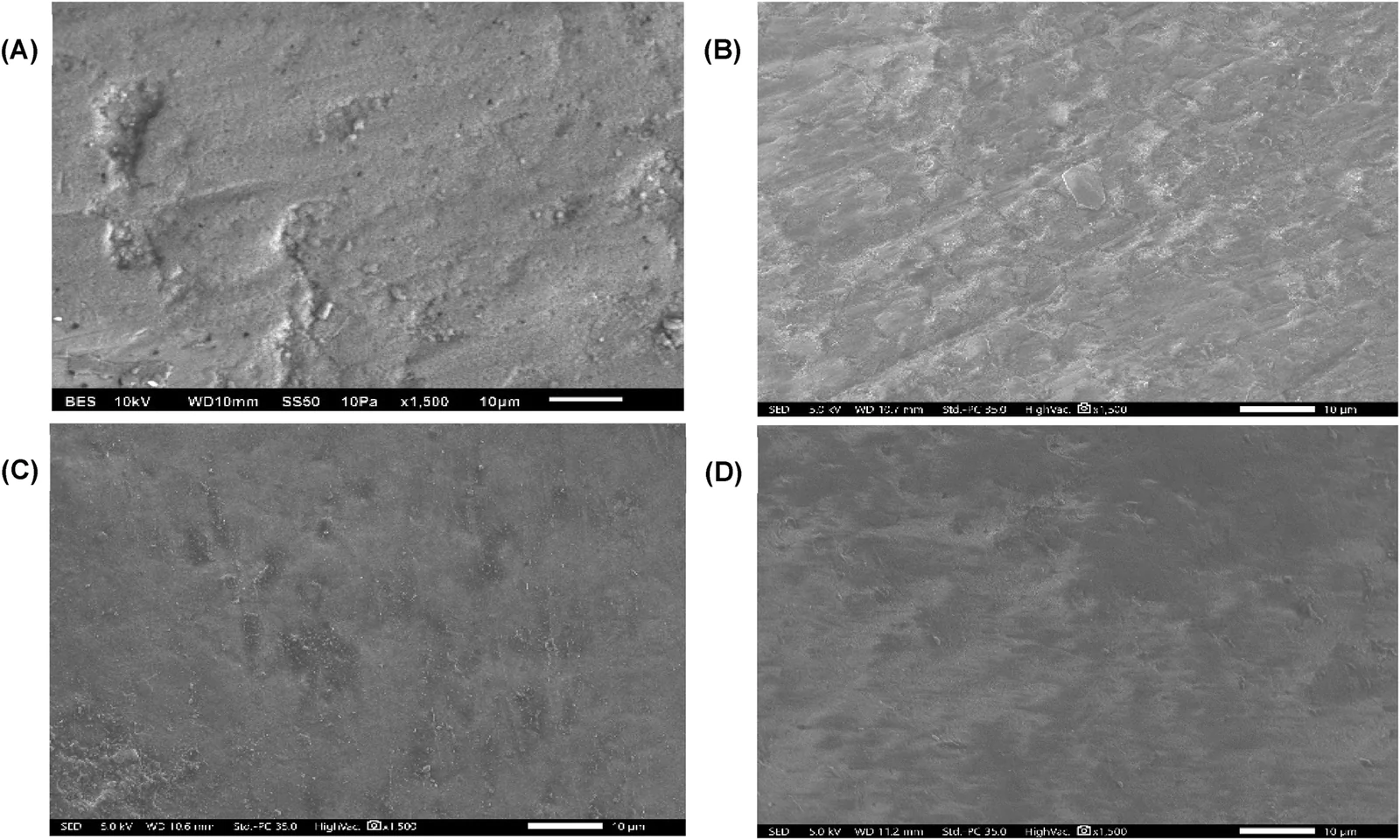
SEM micrographs depicting samples bonded to brackets (G1), **(A)** T0 without treatment, **(B)** T60 stored in artificial saliva (G1a), **(C)** T60 saliva + nHAp (G1b), **(D)** T60 deionized water + nHAp (G1c).

The SEM micrographs in group G1 revealed a surface with irregular elevations and depressions, exposing enamel prisms. In group 1b, a smoother surface was observed, characterized by tubule obliteration (Figure 2).

**Figure 3:**
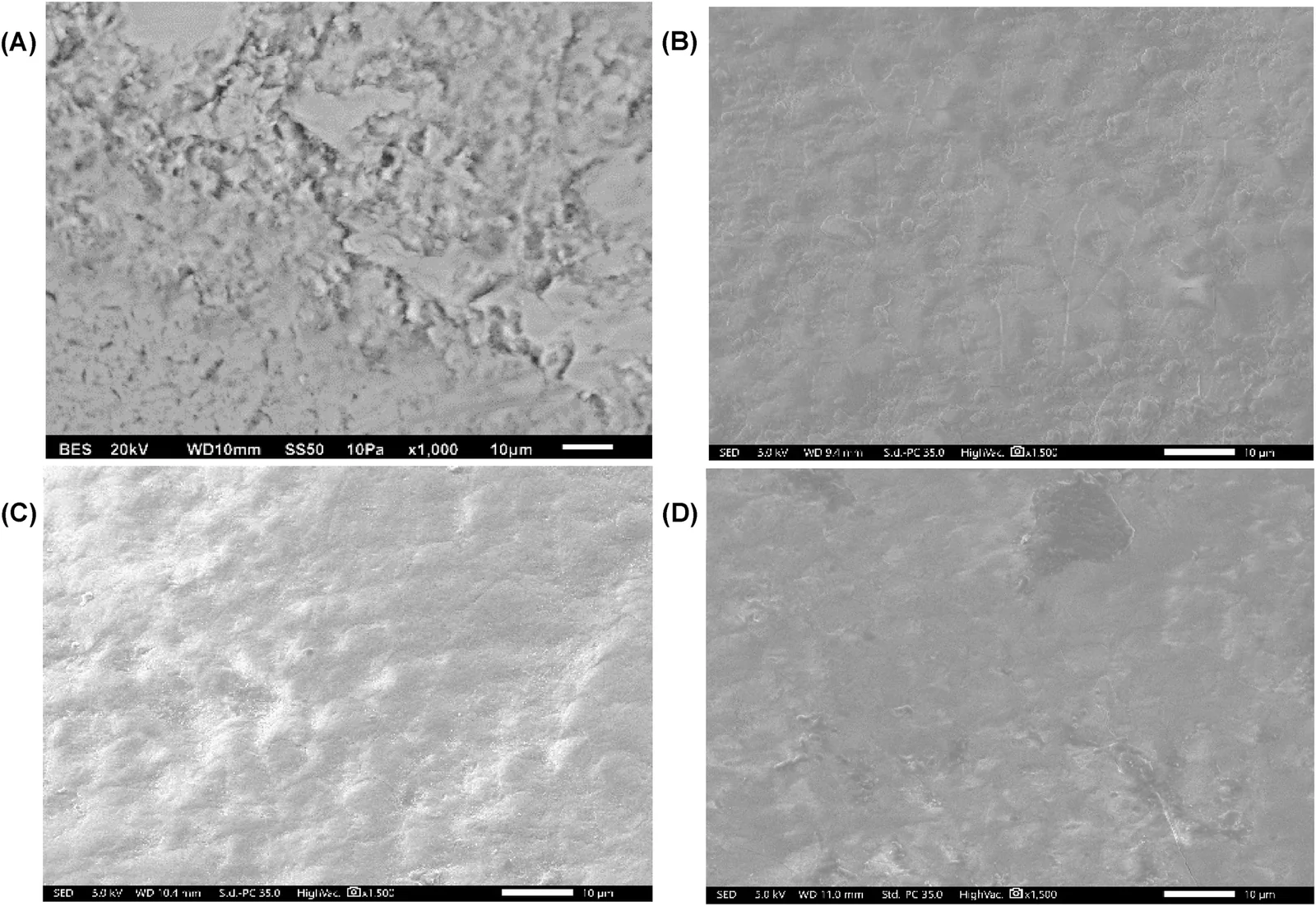
SEM micrographs depicting samples of the bracket laser group (G2), **(A)** T0 without treatment, **(B)** T60 stored in artificial saliva (G2a), **(C)** T60 saliva + nHAp (G2b), **(D)** T60 deionized water + nHAp (G2c).

In group G2, the surface initially revealed an irregular pattern with micro-reliefs and depressions. After immersion in saliva, the surface appeared smoother, and this morphology remained even after the application of nHAp paste (Figure 3).

**Figure 4:**
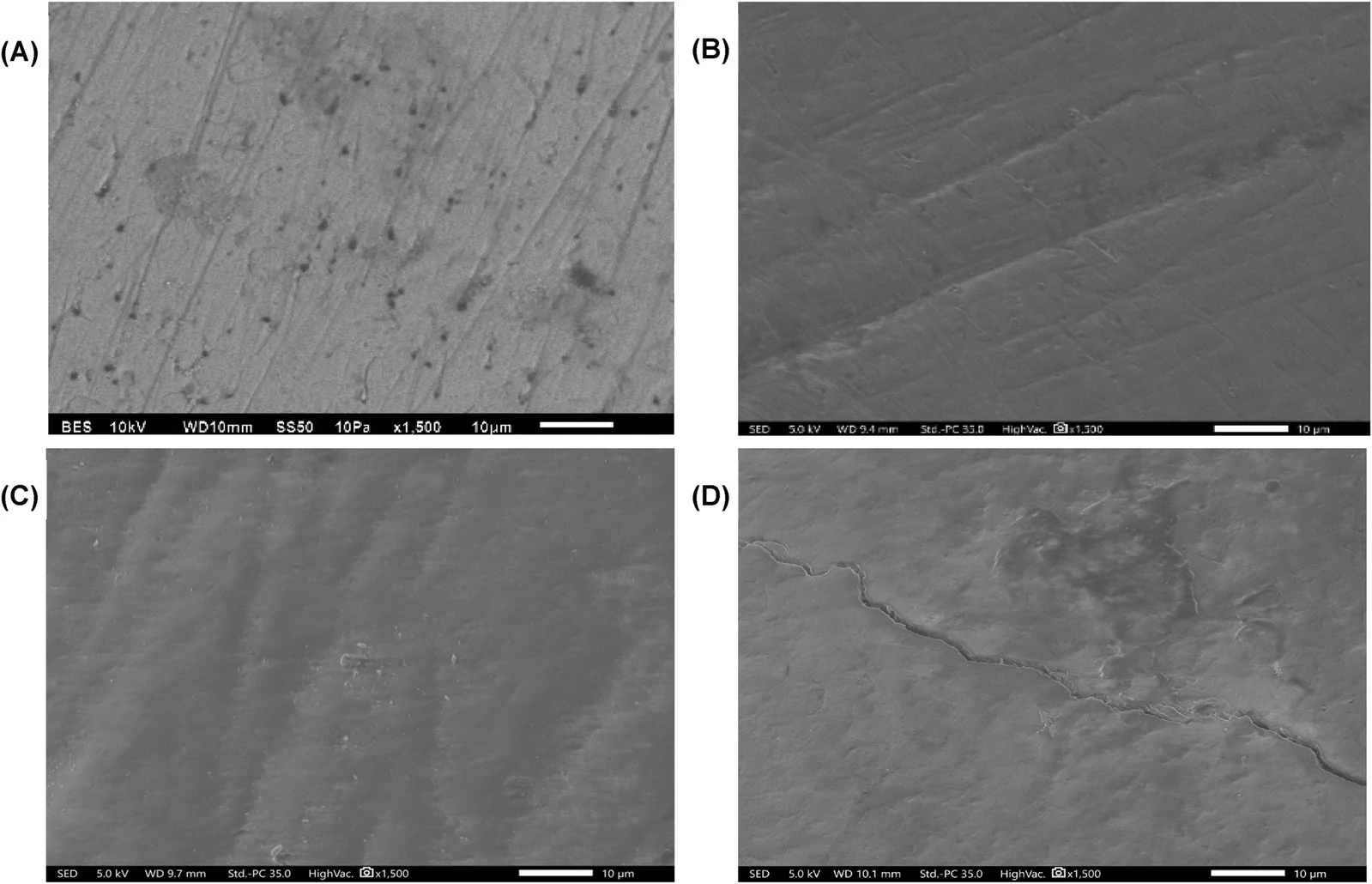
SEM micrographs depicting polished enamel (G3) samples at different storage conditions, **(A)** T0 without treatment, **(B)** T60 stored in artificial saliva (G3a), **(C)** T60 saliva + nHAp (G3b), **(D)** T60 deionized water + nHAp (G3c).

An irregular pattern with marked lines as well as the classic hexagon pattern with the presence of porosities similar to healthy enamel was shown in group G3 (Figure 4). After immersion in saliva, a softened surface was observed; however, it still maintained the presence of scratches, these being to fade in the group where the nHAp paste was applied, showing a much more homogeneous and smoother surface.

**Figure 5:**
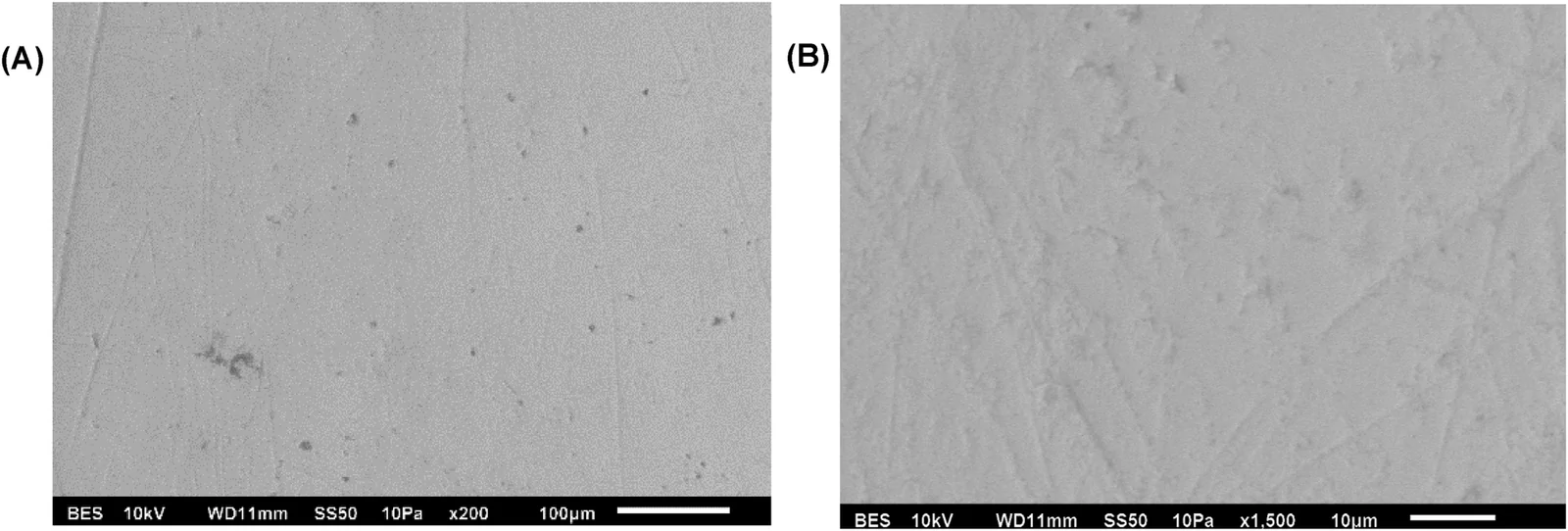
SEM micrographs of healthy enamel (G4). (**A**) enamel surface at x200, (**B**) x1500.

The images obtained in group G4 show microtextured surfaces with the appearance of dotting; overall, a smooth surface was observed (Figure 5).

## 4 Discussion

The primary goal of any orthodontic treatment is to establish structural, functional, and aesthetic harmony between the teeth and the occlusion. Once the treatment with fixed appliances is completed, the attached devices are removed. However, the enamel surface gets damaged, so it must be rehabilitated to restore it as fully as possible to its previous state. Nevertheless, the process of bracket removal and subsequent enamel polishing has raised clinical concerns, since it can compromise the integrity of the enamel structure (21). This issue has prompted an intense search for safe and effective methods to prevent this, leading to the development and implementation of various specialized tools and techniques (1, 28, 29).

In response to these limitations, laser technology has emerged as an effective and minimally invasive alternative for debonding aesthetic brackets. CO_2_ laser has gained clinical relevance due to its thermal softening mechanism, which facilitates controlled bracket debonding without excessive mechanical stress on the enamel (21, 30, 31). In our study, group G2 demonstrated the lowest debonding forces using CO_2_ laser at 6 W for 5 s. Furthermore, Macri et al. (32) achieved greater SBS reduction with 10 W. They also confirmed pulpal safety with temperature increases remaining below the critical threshold of 5.5 °C. Finally, an ARI of 2 was observed, so they concluded that CO_2_ laser was a safe alternative for debonding. In our case, adhesive fracture occurred at the enamel-resin interface (ARI 1), which is clinically favorable, as it reduces enamel damage and facilitates safer adhesive removal and polishing, in line with findings by Saito et al. (33), who reported no significant changes in ARI values.

The efficacy of CO_2_ laser is attributed to the alignment of its wavelengths (9.3–10.6 μm) with the absorption bands of phosphate, carbonate, and hydroxyl groups in enamel and dentin. This interaction enables structural modification without inducing thermal damage (23, 34).

The removal of orthodontic brackets represents a critical phase in treatment, as it may compromise enamel surface integrity if inappropriate techniques are employed. The extent of enamel damage is influenced by the type of instrument used for bracket debonding and residual adhesive removal (35, 36). In the present study, tungsten carbide burs and aluminum oxide tips were selected, as previous research has identified these tools as being the least aggressive alternatives in conventional debonding procedures. Thawaba et al. (35) reported that tungsten carbide burs produced varied surface markings, including deep scratches; however, based on the Enamel Surface Index (ESI), the resulting surfaces were deemed acceptable. These findings align with our SEM observations in group G1, where pronounced enamel irregularities were evident, likely due to the hardness of the bur relative to the adhesive and enamel.

In contrast, prior studies have confirmed that CO₂ laser reduces adhesive bond strength while preserving enamel morphology, yielding roughness values comparable to conventional polishing (10, 30). Our results corroborate these findings, as groups G3a and G2a exhibited similar mean roughness values, whereas group G1a showed significantly increased roughness.

Complementary evidence from Vidor et al. (29) indicated smooth enamel surfaces following bracket debonding and resin removal with aluminum oxide tips, although their study lacked quantitative roughness data. In contrast, our profilometric analysis revealed that group G1 exhibited the highest roughness values, with surface depressions and a characteristic honeycomb enamel pattern. This group also showed a predominance of ARI scores 2 and 3, consistent with our findings.

Despite these advancements, residual roughness following debonding remains a clinical concern, not only due to bracket removal but also because of the subsequent enamel treatment required to eliminate adhesive remnants, particularly when using carbide burs (36, 37).

In this context, nHAp has been investigated as a remineralizing agent capable of repairing microscopic enamel defects (38, 39). In our study, samples stored in artificial saliva without nHAp application showed stable or improved roughness, whereas those stored in deionized water exhibited progressive roughness increases, even with nHAp treatment. This may be attributed to the absence of minerals and buffering agents that mitigate the acidifying effects of the medium (40). SEM analysis of group G2c revealed a partially smooth surface with localized enamel irregularities.

According to the results obtained in this study, the combination of CO_2_ laser and nHAp did not yield significant reductions in surface roughness. The only group showing sustained roughness reduction was the G1 treated with nHAp, particularly in samples stored in saliva. Ra values decreased at 15 days, while Rq and Rz parameters improved at 60 days, suggesting a potential interaction between nHAp and salivary components. This may be due to the ability of nHAp nanoparticles (50–1000 nm) to penetrate enamel pores and act as nucleation sites for calcium and phosphate ions, promoting remineralization (41) in synergy with the salivary buffering system.

These findings are consistent with Verma et al. (14), who reported significant roughness reduction following 15 days of brushing with nHAp post-debonding. However, Ajami et al. (42) found no statistically significant changes in roughness after 10 and 60 days of nHAp serum or toothpaste application and observed irreversible enamel surface alterations in both study groups. Vicente et al. (37) emphasized the need to protect enamel post-debonding, citing SEM evidence of enamel loss and increased susceptibility to demineralization compared to intact enamel.

Ultimately, while CO_2_ laser and nHAp demonstrated individual benefits under the conditions of this *in-vitro* study, their combined application did not reveal a synergistic effect. This limits their consideration as a complementary strategy at the current stage. Nonetheless, their selective use in specific contexts may hold potential for enhancing outcomes by minimizing iatrogenic damage and supporting enamel recovery, although further clinical validation is required. In addition, intrapulpal temperature changes were not measured, which represents a limitation regarding the assessment of thermal safety during CO₂ laser irradiation in this study.

## 5 Conclusions

The CO_2_ laser showed potential for debonding ceramic brackets by reducing forces and preserving enamel morphology. At the same time, nHAp also demonstrated promise for repairing post-treatment defects in conventional bracket debonding.

## Data Availability

The original contributions presented in the study are included in the article/Supplementary Material, further inquiries can be directed to the corresponding author.

## References

[1] Tawfik MA Elgamily M Shamaa MS. Impact of clear aligners and different esthetic orthodontic brackets on enamel demineralization (A comparative SEM study). Egypt Dent J. (2024) 70:3141–8. 10.21608/edj.2024.311672.3164

[2] Borges L Castro ACR Elias CN Souza MMG. Effect of cigarette smoke on aesthetic brackets: an in vitro study. Dental Press J Orthod. (2022) 27:e2220365. 10.1590/2177-6709.27.4.e2220365.oar 36074431 PMC9439572

[3] Ansari MY Gupta A Bhattacharya P Ansar J Bhandari R. Shear bond strength of ceramic brackets with different base designs: comparative in-vitro study. J Clin Diagn Res. (2016) 11:ZC64–8. 10.7860/JCDR/2016/20624.8910 PMC5198460 28050507

[4] Grosch K Meister J Raval SD Fouda AM Bourauel C. Comparative evaluation of different debonding and reconditioning methods for orthodontic ceramic brackets regarding effectiveness for reuse. J Orofac Orthop. (2025) 86:11–23. 10.1007/s00056-023-00469-z 37318554 PMC11753313

[5] Delavarian M Rahimi F Mohammadi R Imani M. Shear bond strength of ceramic and metal brackets bonded to enamel using color-change adhesive. Dent Res J. (2019) 16:233. 10.4103/1735-3327.261128 PMC6596176 31303877

[6] Espinosa IA Katagiri MK Ibarra GJ. Estudio comparativo de la fuerza de adhesión de brackets policristalinos de adhesión química y monocristalinos de adhesión mecánica. Rev Odontol Mex. (2004) 8(1-2):7–9. 10.22201/fo.1870199xp.2004.8.1-2.16116

[7] Zamzam MK Hamadah O Espana-Tost T Arnabat-Dominguez J. Comparative study of transmission of 2940 nm wavelength in six different aesthetic orthodontic brackets. Dent J. (2023) 11:67. 10.3390/dj11030067 PMC10047171 36975563

[8] Russell JS. Aesthetic orthodontic brackets. J Orthod. (2005) 32:146–63. 10.1179/146531205225021024 15994990

[9] Woś P Kiryk S Dyl T Kiryk J Horodniczy T Szablińska M. Laser applications in metal orthodontic bracket debonding: a systematic review. Appl Sci. (2025) 15:927. 10.3390/app15020927

[10] Ferreira LJT Borsatto MC Pereira SMC Matsumoto NMA Torres CP Romano FL. Evaluation of enamel roughness in vitro after orthodontic bracket debonding using different methods of residual adhesive removal. Turk J Orthod. (2020) 33:43–51. 10.5152/TurkJOrthod.2020.19016 32284898 PMC7138237

[11] Almudhi A Aldeeri A Aloraini AAA Alomar AIM Alqudairi MSM Alzahrani OAA. Comparison of enamel surface integrity after de-bracketing as affected by seven different orthodontic residual cement removal systems. Diagnostics. (2023) 13:3284. 10.3390/diagnostics13203284 37892104 PMC10606188

[12] Saudi R Ibrahim MA. Comparison between nano-hydroxyapatite and CPP-ACPF in remineralizing early carious lesions (in vitro study). BAU J Creat Sustain Dev. (2020) 2:1–11. 10.54729/2789-8334.1031

[13] Vijayasankari V Asokan S GeethaPriya PR. Evaluation of remineralisation potential of experimental nano hydroxyapatite pastes using scanning electron microscope with energy dispersive x-ray analysis: an in-vitro trial. Eur Arch Paediatr Dent. (2019) 20:529–36. 10.1007/s40368-018-00411-7 30904993

[14] Verma P Pandian MS. Bionic effects of nano hydroxyapatite dentifrice on demineralized surface of enamel post orthodontic debonding: in-vivo split mouth study. Prog Orthod. (2021) 22:39. 10.1186/s40510-021-00381-5 34719755 PMC8558117

[15] Ghazanfari R Nokhbatolfoghahaei H Alikhasi M. Laser-aided ceramic bracket debonding: a comprehensive review. J Lasers Med Sci. (2016) 7:2–11. 10.15171/jlms.2016.02 27330690 PMC4908986

[16] Xavier J Sarika K Ghosh P Varma S. Aesthetic bracket system: a review. Int J Dent Oral Sci. (2021) 12:5191–6. 10.19070/2377-8075-210001041

[17] Abdulmajeed A Phan T Grzech-Lesniak K Deeb JG. An in vitro study comparing debonding of orthodontic ceramic and metal brackets using er:yAG laser and conventional pliers. Appl. Sci. (2025) 21:11844. 10.3390/app152111844

[18] Khalil AS Tamish NM Elkalza AR. Assessment of chemical, ultrasonic, diode laser, and Er:YAG laser application on debonding of ceramic brackets. BMC Oral Health. (2022) 22:79. 10.1186/s12903-022-02111-7 35305631 PMC8933975

[19] Florczak-Matyjek A Nikodem A Kensy J Matys J Grzech-Leśniak K. The efficacy of erbium-ion, diode, and CO_2_ lasers in debonding attachments used during overlay orthodontic treatment and the risk of hard tooth tissue damage compared to traditional methods—an in vitro study. Photonics. (2025) 12:621. 10.3390/photonics12060621

[20] Downarowicz P Noszczyk P Mikulewicz M Nowak R. Thermal effect of Er:YAG and er,cr:YSGG used for debonding ceramic and metal orthodontic brackets: an experimental analysis. Adv Clin Exp Med. (2020) 29:557–63. 10.17219/acem/118844 32396714

[21] Michalak M Kiryk S Kotela A Wiśniewska K Kiryk J Zborowski JZ. Orthodontic ceramic bracket removal using lasers: a systematic review. J Funct Biomater. (2025) 16:123. 10.3390/jfb16040123 40278231 PMC12027597

[22] Ahrari F Boruziniat A Alirezaei M. Surface treatment with a fractional CO_2_ laser enhances shear bond strength of resin cement to zirconia. Laser Ther. (2016) 25:19–26. 10.5978/islsm.16-OR-01 PMC4846836 27141151

[23] Luk K Zhao IS Gutknecht N Chu CH. Use of carbon dioxide lasers in dentistry. Lasers Dent Sci. (2019) 3:1–9. 10.1007/s41547-018-0047-y

[24] Ekici Ö Aslantaş K Kanık Ö Keleş A. Evaluation of surface roughness after root resection: an optical profilometer study. Microsc Res Tech. (2021) 84:828–36. 10.1002/jemt.23714 33491839

[25] Kim J Shin W. How to do random allocation (randomization). Clin Orthop Surg. (2014) 6:103–9. 10.4055/cios.2014.6.1.103 24605197 PMC3942596

[26] International Organization for Standardization. Geometrical Product Specifications (GPS) — Surface Texture: Profile — Part 2: Terms, Definitions and Surface Texture Parameters. Geneva: ISO (2021).

[27] International Organization for Standardization. Geometrical Product Specifications (GPS) — Surface Texture: Profile — Part 3: Rules and Procedures for the Assessment of Surface Texture. Geneva: ISO (2021).

[28] Soares Tenório KC Neupmann Feres MF Tanaka CJ Augusto MKM Rodrigues JA Pereira da Silva HD. In vitro evaluation of enamel surface roughness and morphology after orthodontic debonding: traditional cleanup systems versus polymer bur. Int Orthod. (2020) 18:546–54. 10.1016/j.ortho.2020.04.006 32493624

[29] Vidor MM Felix RP Marchioro EM Hahn L. Enamel surface evaluation after bracket debonding and different resin removal methods. Dental Press J Orthod. (2015) 20:61–7. 10.1590/2176-9451.20.2.061-067.oar 25992989 PMC4445227

[30] Matos DS Küchler EC Borsatto MC Matsumoto MAN Marques FV Romano FL. CO_2_ laser irradiation for debonding ceramic orthodontic brackets. Braz Dent J. (2021) 32:45–52. 10.1590/0103-6440202104077 34614060

[31] Moshkelgosha V Zandian R Sohrabi M Fekrazad R. Effect of CO_2_ laser-assisted titanium tetra-fluoride on demineralization of enamel around orthodontic brackets. J Lasers Med Sci. (2024) 15:e9. 10.34172/jlms.2024.09 39050994 PMC11267097

[32] Macri RT de Lima FA Bachmann L Galo R Romano FL Borsatto MC. CO_2_ laser as auxiliary in the debonding of ceramic brackets. Lasers Med Sci. (2015) 30:1835–41. 10.1007/s10103-014-1688-z 25410302

[33] Saito A Namura Y Isokawa K Shimizu N. CO_2_ laser debonding of a ceramic bracket bonded with orthodontic adhesive containing thermal expansion microcapsules. Lasers Med Sci. (2015) 30:869–74. 10.1007/s10103-013-1482-3 24220847

[34] Silva CV Mantilla TF Engel Y Tavares JP Freitas PM Rechmann P. The effect of CO_2_ 9.3 μm short-pulsed laser irradiation in enamel erosion reduction with and without fluoride applications—a randomized, controlled in vitro study. Lasers Med Sci. (2020) 35:1213–22. 10.1007/s10103-020-02979-3 32030555

[35] Thawaba AA Albelasy NF Elsherbini AM Hafez AM. Evaluation of enamel roughness after orthodontic debonding and clean-up procedures using zirconia, tungsten carbide, and white stone burs: an in vitro study. BMC Oral Health. (2023) 23:478. 10.1186/s12903-023-03194-6 37443027 PMC10339551

[36] D'Amario M Bernardi S Di Lauro D Marzo G Macchiarelli G Capogreco M. Debonding and clean-up in orthodontics: evaluation of different techniques and micro-morphological aspects of the enamel surface. Dent. J. (2020) 2(58):1–10. 10.3390/dj8020058 PMC7344892 32560482

[37] Vicente A Ortiz Ruiz AJ García López M Martínez Beneyto Y Bravo-González LA. Enamel resistance to demineralization after bracket debonding using fluoride varnish. Sci Rep. (2017) 7:15183. 10.1038/s41598-017-15600-5 29123323 PMC5680324

[38] Jardim RN Rocha AA Rossi AM de Almeida Neves A Portela MB Lopes RT. Fabrication and characterization of remineralizing dental composites containing hydroxyapatite nanoparticles. J Mech Behav Biomed Mater. (2020) 109:1–10. 10.1016/j.jmbbm.2020.103817 32543392

[39] Sahin D Deger C Oglakci B Demirkol M Kucukyildirim BO Gursel M. The effects of a novel nanohydroxyapatite gel and Er: YAG laser treatment on dentin hypersensitivity. Materials (Basel). (2023) 16:6522. 10.3390/ma16196522 37834658 PMC10573567

[40] Akbeyaz E Parlakyıldız AN Kargül B. Effect of different remineralization agents on artificial caries lesion: an in-vitro study. Clin Exp Health Sci. (2023) 13:330–6. 10.33808/clinexphealthsci.1103037

[41] Rajendran R Antony S DP Ashik P M Bharath S Thomas AJ Heboyan A. Remineralization potential of strontium-doped nano-hydroxyapatite dentifrice and casein phosphopeptide-amorphous calcium phosphate cream on white spot lesions in enamel following orthodontic debonding – a randomized controlled trial. SAGE Open Med. (2024) 12:1–9. 10.1177/20503121231221634 PMC10757435 38162913

[42] Ajami S Pakshir HR Babanouri N. Impact of nanohydroxyapatite on enamel surface roughness and color change after orthodontic debonding. Prog Orthod. (2016) 17:11. 10.1186/s40510-016-0124-2 27004806 PMC4826863

